# Dental divisions: exploring racial inequities of dental caries amongst children

**DOI:** 10.1038/s41432-024-00977-w

**Published:** 2024-01-26

**Authors:** Sean Daley, Anna Nugent, Greig D. Taylor

**Affiliations:** 1 Newcastle Dental Hospital, Newcastle Upon Tyne, UK; 2 Newcastle University, Newcastle Upon Tyne, UK

**Keywords:** Dental caries, Paediatric dentistry

## Abstract

**Data sources:**

The search strategy involved three sequential stages. Initially, MEDLINE/PubMed was explored for relevant articles, identifying pertinent terms for formal searching. Using the terms ethnic, race, minoritised and dental caries, a strategy was formed and nine databases searched. Finally, hand-searching of reference lists of included articles and sourcing grey literature from relevant government reports, national oral health surveys, and registries which had comparative data for dental caries between racial groups, completed the search.

**Study selection:**

Studies included were original primary research which reported dental caries and compared racially minoritised children, aged 5–11 years, to similarly aged from national, majority, or privileged populations. Dental caries had to be recorded from a clinical examination which assessed decayed, missing, and filled teeth (dmft) in primary dentitions. Studies were excluded if they used immigration status as a basis of racial status, or they were a case report, case series, in vitro study, or literature review.

**Data extraction and synthesis:**

After removing duplicates, two independent researchers screened abstracts, prior to extracting critical data following full-text reviews of included articles. Information collected included study and participant characteristics, definitions of race, and dental caries measurement. The authors of studies which had missing data were contacted, whilst those not written in the English language were translated. Methodological quality of each study was independently assessed by two reviewers using a modified version of the Newcastle-Ottawa scale. All studies were included in the review regardless of quality. A narrative overview of all included studies was conducted. Meta-analyses were completed using studies that reported the mean and standard deviation of the caries outcomes in both groups. Caries outcomes included severity (defined as mean dmft) or prevalence (percentage of teeth with untreated dental caries > 0%). Due to anticipated heterogeneity, statistical analyses approaches such as I^*2*^ statistics were used to estimate between-study variability. Additional sub-group analyses were conducted based on country of study and world income index. Contour-enhanced funnel plots and trim-and-fill analysis were completed to explore potential publication bias. Sensitivity analyses were performed to ensure robustness of the findings.

**Results:**

Seventy-five studies were included from a variety of countries. A higher mean dmft score of 2.30 (0.45, 4.15) and prevalence of decayed teeth (*d* > 0) was 23% (95% CI: 16, 31) was noted amongst racially minoritised children compared to privileged children’s populations. Notable disparities were reported in high-income countries, with minoritised children burdening the greatest distribution of caries incidence. The study faced challenges in consistent racial classification and encountered high heterogeneity in its findings, leading to varied GRADE assessment scores.

**Conclusions:**

The study calls for global, social, and political changes to tackle the substantial disparities in dental caries among minoritised children to achieve oral health equity.

## A Commentary on

**Nath S, Sethi S, Bastos J L et al.**

The global prevalence and severity of dental caries among racially minoritised children: a systematic review and meta-analysis. *Caries Res* 2023; **57:** 485–508.

**GRADE Rating:** 

## Commentary

Our GRADE rating for this review of ‘High’ can be justified by several factors. Strict adherence to PRISMA guidelines meant a strong methodological framework was adopted. Comprehensive data sources were utilised, ensuring a wide range of studies, irrespective of language, were included. A detailed, systematic study selection was adopted^1^. The study’s data extraction and synthesis methods employed qualitative and quantitative analyses with appropriate subgroup analyses^2^ and sensitivity tests^3,4^. The results provided clear, quantifiable disparities in dental caries among racially minoritised children, particularly in high-income countries with a growing trend over time. The methodological rigour and comprehensive nature of this review lends high confidence in the reliability and relevance of its findings.

This study is a comprehensive and methodologically sound exploration of a critical global public health issue. It illuminates the significant disparities in dental health faced by racially minoritised children. Barriers faced to improved oral health include limited access to dental care, financial and language constraints. As such the FDI World Dental Federation and the World Health Organistaion have created initiatives to limit the oral health burden to these groups. Recognition that oral health is a key indicator of overall health is paramount and aligns with their goals of health equity^5^. Despite its methodological strengths, the study relies on cross-sectional data, which restricts causal inferences. Longitudinal studies could be recommended for future research to overcome this issue. This would enable understanding of the progression of oral health disparities over time, offering insights into the effectiveness of current interventions and permitting more nuanced analysis of how external factors contribute to inequalities, enabling the development of more effective, enduring solutions. Predominance of studies from high-income countries may limit the universality of the findings, and efforts should be made to ensure data from low-income countries are captured in future research. In addition, classification of race and ethnicity varies across studies, which has the potential to affect the consistency of the data. Despite these limitations, the study provides valuable insights into dental health disparities among racially minoritised children.

Practice points

- The findings underscore the need for targeted public health interventions and policies that address the broader societal and structural factors contributing to dental health disparities among racially minoritised populations.
- The use of these findings in each country will ensure approaches are pertinent to their populations, keeping cultural relevancy at the forefront.
- To maximise effectiveness, the views of culturally minoritised populations need to be considered to ensure sensitivity to issues. Only by including this and utilising reviews, can we make positive changes at a global level.
